# Functional characterization of enhancer evolution in the primate lineage

**DOI:** 10.1186/s13059-018-1473-6

**Published:** 2018-07-25

**Authors:** Jason C. Klein, Aidan Keith, Vikram Agarwal, Timothy Durham, Jay Shendure

**Affiliations:** 1 Department of Genome Sciences, University of Washington, Seattle, WA 98195 USA; 2 Howard Hughes Medical Institute, University of Washington, Seattle, WA 98195 USA; 3 Brotman Baty Institute for Precision Medicine, Seattle, WA 98195-8047 USA

## Abstract

**Background:**

Enhancers play an important role in morphological evolution and speciation by controlling the spatiotemporal expression of genes. Previous efforts to understand the evolution of enhancers in primates have typically studied many enhancers at low resolution, or single enhancers at high resolution. Although comparative genomic studies reveal large-scale turnover of enhancers, a specific understanding of the molecular steps by which mammalian or primate enhancers evolve remains elusive.

**Results:**

We identified candidate hominoid-specific liver enhancers from H3K27ac ChIP-seq data. After locating orthologs in 11 primates spanning around 40 million years, we synthesized all orthologs as well as computational reconstructions of 9 ancestral sequences for 348 active tiles of 233 putative enhancers. We concurrently tested all sequences for regulatory activity with STARR-seq in HepG2 cells. We observe groups of enhancer tiles with coherent trajectories, most of which can be potentially explained by a single gain or loss-of-activity event per tile. We quantify the correlation between the number of mutations along a branch and the magnitude of change in functional activity. Finally, we identify 84 mutations that correlate with functional changes; these are enriched for cytosine deamination events within CpGs.

**Conclusions:**

We characterized the evolutionary-functional trajectories of hundreds of liver enhancers throughout the primate phylogeny. We observe subsets of regulatory sequences that appear to have gained or lost activity. We use these data to quantify the relationship between sequence and functional divergence, and to identify CpG deamination as a potentially important force in driving changes in enhancer activity during primate evolution.

**Electronic supplementary material:**

The online version of this article (10.1186/s13059-018-1473-6) contains supplementary material, which is available to authorized users.

## Background

Despite seemingly large phenotypic differences between species across the primate lineage, protein-coding sequences remain highly conserved. Britten and Davidson as well as King and Wilson proposed that changes in gene regulation account for a greater proportion of phenotypic evolution in higher organisms than changes in protein sequence [1, 2]. A few years later, Banerji and Moreau observed that the SV40 DNA element could increase expression of a gene independent of its relative position or orientation to the transcriptional start site [3, 4]. This finding led to the characterization of a new class of regulatory elements, enhancers.

Several aspects of enhancers make them ideal substrates for evolution. Enhancers control the location and level of gene expression in a modular fashion [5]. While a coding mutation will disrupt function throughout an organism, a mutation in an enhancer may only affect the expression of a gene at a particular time and location. This modularity of regulatory elements may facilitate the development of novel phenotypes, e.g. by decreasing pleiotropy [6]. Enhancers also commonly exist in groups of redundant elements, referred to as shadow enhancers, which provide phenotypic robustness [7–9]. Therefore, mutations within enhancers generally exhibit lower penetrance than mutations in coding sequences, facilitating the accumulation of variation.

Researchers have studied the role of enhancers in evolution through two main methods: high-resolution, systematic analysis of single enhancers, or low-resolution, genome-wide analysis of many enhancers. Examples of the former include fruitful investigations of how specific enhancers underlie phenotypic changes, e.g. cis-regulatory changes of the *yellow* locus affecting Drosophila pigmentation [10, 11], recurrent deletions of a *Pitx1* enhancer resulting in the loss of pelvic armor in stickleback [12], and recurrent SNPs in the intron of *MCM6,* resulting in lactase persistence in humans [13, 14].

Low-resolution, genome-wide approaches for discovering candidate enhancers via biochemical marks, when applied to multiple species, have identified large-scale turnover of enhancers between human and mouse embryonic stem cells [15], human and mouse preadipocytes and adipocytes [16], mammalian limb bud [17], and vertebrate and mammalian liver [18, 19].

Low-resolution studies have the advantage of characterizing thousands of enhancers at a time, but fail to pinpoint functional variation. In contrast, high-resolution studies can provide clear insights into the evolution of individual enhancers, but the findings may not be broadly generalizable. Applying massively parallel reporter assays (MPRAs) to a closely related phylogeny may offer an opportunity to bridge the insights offered by low- and high-resolution studies. MPRAs have enabled high-resolution functional dissection of enhancers by testing the effects of naturally occurring and synthetic variation on regulatory activity since their inception [20–25], but have only recently been applied to study enhancer evolution [26, 27]. For example, STARR-seq was used to characterize enhancer evolution within five Drosophila species, providing functional evidence of large-scale turnover [26].

Here we set out to concurrently study the evolutionary-functional trajectories of hundreds of enhancers with MPRAs. We identified potential hominoid-specific liver enhancers based on genome-wide ChIP-seq and then functionally tested all of these in parallel. After identifying “active tiles” of these candidate enhancers, we tested eleven primate orthologs and nine predicted ancestral reconstructions of each active tile for their relative activity. Normalizing to the activity of the reconstructed sequences of the common ancestor of hominoids and Old World monkeys, we identify several subsets of active tiles that appear to have gained or lost activity along specific branches of the primate lineage; only some of these patterns are consistent with ChIP-seq-based expectations. We also use these data to examine how the accumulation of mutations impacts enhancer activity across the phylogeny, quantifying the correlation between sequence divergence and functional divergence. Finally, we examine the set of mutations that appear to drive functional changes, and find enrichment for cytosine deamination within CpGs.

## Results

### Identification of candidate hominoid-specific enhancers

**Fig. 1:**
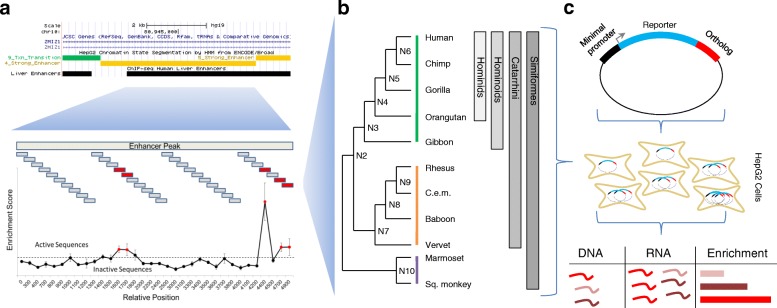
Schematic of Experimental Design. **a** We identified potential hominoid-specific enhancers by intersecting hominoid-specific ChIP-seq predicted enhancers from primary human liver with ChromHMM-predicted strong enhancers in HepG2 cells (screenshot from http://genome.ucsc.edu) [54]. We then tiled across each candidate enhancer using 194 nt sequences and identified 697 tiles that were active in the STARR-seq reporter assay in HepG2 cells. **b** We located orthologous sequences in 11 primates and computationally reconstructed 9 ancestral sequences for 348 of the active tiles, using New World monkeys as an outgroup. **c** We then cloned all 20 present-day or ancestral orthologs per tile and performed STARR-seq again in HepG2 cells. After collecting DNA and RNA from cells, we calculated enrichment scores as the log_2_ ratio of RNA to DNA for each ortholog. Each shade of red represents a different ortholog tested

From a published ChIP-seq study in mammals [18, 28], we identified 10,611 H3K27ac peaks (associated with active promoters and enhancers) that were present in humans and absent from macaque to tasmanian devil, and that were not within 1 kilobase (kb) of a H3K4me3 peak (associated with active promoters). We considered this set of peaks as potential hominoid-specific enhancers (active within the clade from gibbon to human). We narrowed this to a subset of 1015 candidate enhancers overlapping ChromHMM strong-enhancer annotations in human HepG2 cells [29] that also had orthologous sequences in the genomes of species from human to marmoset. On average, the intersection between the hominoid-specific H3K27ac peak and HepG2 ChromHMM call was 1138 bp (Additional file 1: Figure S1A). In order to identify active subregions of each candidate enhancer, we designed 194 nt sequences tiling across the length of each, overlapping by 93-100 bp (Fig. 1a).

We synthesized and tested all 10,544 tiles for enhancer activity in a massively parallel reporter assay. Specifically, we used the STARR-seq vector [30], in which candidate enhancers are cloned into the 3′ UTR of an episomal reporter gene, in human HepG2 cells in triplicate. After extracting, amplifying, and sequencing DNA and RNA corresponding to the enhancer regions from transfected cells, we calculated an enrichment score for each tile as the log_2_ of the normalized ratio of RNA to DNA (rho for pairs of replicates between 0.581 and 0.676) (Additional file 2: Table S1, Additional file 1: Figure S2). We defined “active tiles” as elements with log_2_ enrichment scores greater than 1. While most of the 1015 candidate enhancers contained no active tiles, we identified 697 active tiles (out of 10,544, or 6.6%), occurring within 34% of the candidate enhancers (Additional file 1: Figure S1B). While we chose a strict cutoff for active tiles to increase specificity, we do note a significant shift towards more positive enrichment scores for all of our tiles as compared to scrambled control sequences (mean score 0.208 v − 0.07, *p* < 1e-5, t-test) (Additional file 1: Figure S1C). We also note enrichments for our active tiles overlapping DHS (1.8-fold), FosL2 ChIP-seq (2.1-fold), JunD ChIP-seq (2.2-fold), and p300 ChIP-seq peaks (1.5-fold) (Fisher’s exact tests, p < 1e-5). While filtering on these marks might boost our ability to predict enhancers, over half of our active tiles did not overlap any of the above marks.

### Computationally predicting the activity of ancestral and orthologous sequences

A goal of this study was to characterize how the number and spectrum of mutations relate to the functional divergence of enhancer activity in primates. We used eleven high-quality primate genomes (human, chimpanzee, gorilla, orangutan, gibbon, rhesus, crab-eating macaque, baboon, vervet, marmoset and squirrel monkey) to locate similarly-sized orthologs of each of our 697 active human tiles. We were able to identify orthologs in all eleven species for 348 of the 697 active human tiles. Since these species are separated by only ~ 40 million years, they retain high nucleotide identity. We sought to take advantage of this to ask whether we could computationally pinpoint the sequence changes that underlie apparent functional differences between orthologous sequences within primates. Of note, we had not yet measured the functional activity of orthologs of active tiles. Rather, we were assuming that previously observed patterns of gain/loss in H3K27 acetylation were reflective of whether particular tiles were active or inactive in each primate.

We first examined turnover of motifs of transcription factors (TFs) known to be associated with enhancer activity in HepG2: FosL2 and JunD [31]. We focused on comparing the human ortholog to the marmoset ortholog, the furthest outgroup with ChIP-seq data. We identified a modest enrichment of the AP-1 consensus motif, the motif for JunD and FosL2 binding, in the human ortholog compared to marmoset (*p* = 0.012, Fisher’s exact). However, AP-1 site turnover could only explain 5% of the gain-of-activity events predicted by H3K27ac ChIP-seq. For a more global analysis, we scanned our human and marmoset ortholog sequences for matches to the HOCOMOCO v9 motif database [32] using FIMO [33] and identified an enrichment in the human orthologs for hepatocyte nuclear factors (2.1-fold, Fisher’s exact *p* = 0.0013) and FoxA transcription factors (3.9-fold, Fisher’s exact *p* = 1e-4) (Additional file 2: Table S2).

**Fig. 2:**
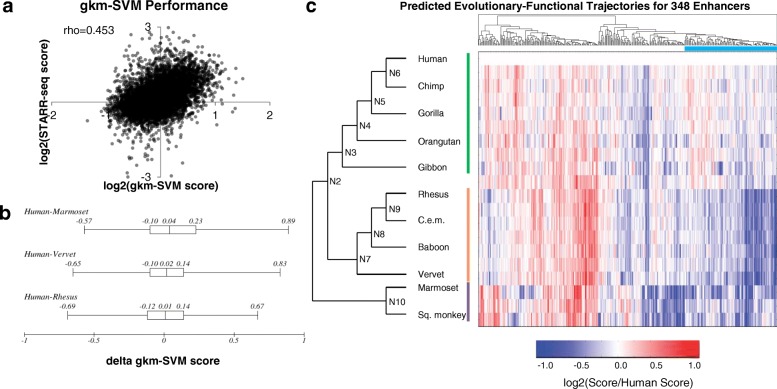
Performance of Computational Predictions. **a** We trained the gapped-kmer support vector machine classifier (gkm-SVM) on an independent reporter assay experiment conducted in HepG2 cells. We then predicted the functional activity of all of our human sequence tiles and found a modest correlation with our functional data. **b** The distributions of differences in predicted gkm-SVM score between the human vs. marmoset, vervet, or rhesus ortholog for all active human tiles. **c** Predicted scores for all orthologs of the 348 human-active enhancer tiles, normalized to the human ortholog. Clades are denoted by colored lines (green: hominoid, orange: Old World monkeys, purple: New World monkeys). Cyan bar below dendrogram denotes a group of 108 enhancer tiles that follows expectations for hominoid-specific enhancers as predicted by ChIP-seq comparative genomics

As a different approach, we built a computational model for predicting enhancer activity in HepG2 cells, and then sought to apply that model to the active tiles and their orthologs. Specifically, we trained a gapped k-mer support vector machine (gkm-SVM), a sequence-based classifier based on the abundance of gapped k-mers in positive and control training data, on an independent massively parallel reporter assay experiment in HepG2 cells [31, 34]. We evaluated the model by predicting the enrichment scores from our tiling experiment on human orthologs, which the model had not seen during training. Although the original data was based on an entirely different MPRA assay (‘lentiMPRA’) and sequences, the scores for each tile predicted from the gkm-SVM model correlated reasonably well with our enrichment scores obtained through STARR-seq in HepG2 cells (Spearman rho = 0.453, *p* < 1e-10) (Fig. 2a, Additional file 2: Table S3). This model outperformed an LS-GKM model trained on a larger dataset of ChIP-seq data from HepG2 [31]. We then used the MPRA-trained model to predict regulatory activity for the rhesus, vervet, and marmoset orthologs, all of which did not have H3K27ac peaks. We expected to find lower predicted activity for these three orthologs compared to human. However, the predicted activity for the human vs. rhesus, vervet, or marmoset orthologs were not significantly different (*p* = 0.10, t-test), although it did trend in the right direction for all three comparisons (Fig. 2b).

With the goal of increasing our power to detect mutations underlying gains or losses in enhancer activity, we reconstructed nine ancestral sequences of the 11 primate orthologs using FastML, a maximum-likelihood heuristic (Fig. 1b) [35]. All ancestral reconstructions except for N10 (most recent common ancestor (MRCA) to New World monkeys) and N2 (MRCA to Catarrhines) had a marginal probability > 0.8 (Additional file 1: Figure S5). We then applied the gkm-SVM model to predict regulatory activities for the 20 orthologs (11 from present-day primate genomes, 9 reconstructed ancestral sequences) of the human-active tiles. To characterize evolutionary trajectories, we performed hierarchical clustering on the vectors of predicted activity for each tile, and identified a group of 108 enhancer tiles that show decreased predicted activity in rhesus, vervet, and marmoset compared to human, following the pattern predicted by H3K27ac ChIP-seq (Fig. 2c). However, this was a clear minority of all tiles evaluated with this computational model (108/348, or 31%). We obtained similar results when using the deltaSVM package with a model trained on HepG2 DNase + H3K4me1 [36].

### Functional characterization of ancestral and orthologous sequences

We were surprised that less than a third of our computational predictions were concordant with ChIP-seq predictions. This could be due to limitations either in interpreting patterns in H3K27ac gain/loss, the computational models that we are applying to predict the relative activities of orthologs, or both. To investigate this further, we synthesized and functionally tested all 20 versions of each of the 348 active tiles with the STARR-seq vector in HepG2 cells. With the goal of improving accuracy and reproducibility, we added degenerate barcodes adjacent to each sequence of interest while cloning the library, so that we could distinguish multiple independent measurements for each element. Furthermore, we performed three biological replicates, which correlated well (independent transfections; Spearman rho between 0.773 and 0.959) (Additional file 1: Figure S3A-C). We took the average enrichment score of all barcodes over all three replicates and filtered out any element with less than six independent measurements. On average, this set had 31 independent measurements per element (Additional file 1: Figure S3D).

**Fig. 3:**
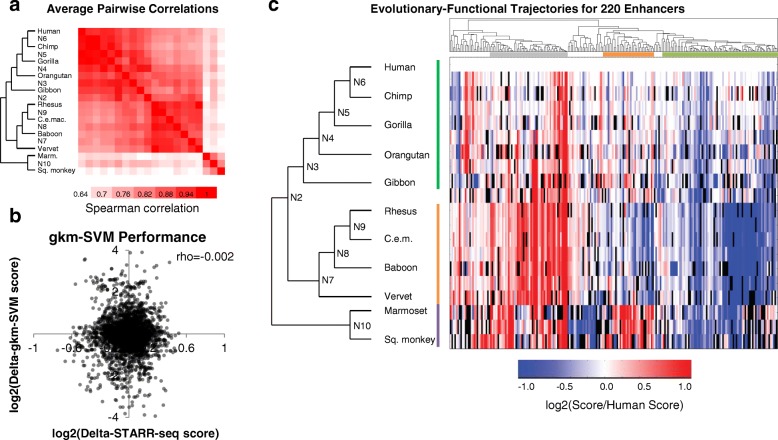
Functional Scores for Orthologs and Ancestral Sequences. **a** The average pairwise Spearman correlation of functional scores between any two orthologs across all enhancer tiles tested. **b** Correlation between the STARR-seq enrichment scores, normalized to the enrichment score of its human ortholog (log_2_[non-human score/human score]), and gkm-SVM predicted scores, similarly normalized to the predicted score of its human ortholog (log_2_[non-human prediction/human prediction]). **c** Functional scores normalized to human for all orthologs of the 220 enhancer tiles. Black bars represent missing data. Clades are denoted by vertical colored lines (green: hominoid, orange: Old World monkeys, purple: New World monkeys). Groups are denoted by horizontal colored lines below the dendrogram; gray: relatively higher activity in in Old World monkeys, orange: relatively lower in Old World monkeys, green: relatively higher activity in either humans or hominoids

The resulting dataset included enrichment scores for 5426 of the 6960 sequences tested (78.0%), corresponding to 344 of the 348 human-active enhancer tiles (98.9%) (Additional file 2: Table S4). As expected, the average pairwise correlation between species was higher within clades (hominoid, Old World monkeys, and New World monkeys) than between clades (Fig. 3a). We do note a lack of correlation between human tiles from our tiling screen and ortholog screen (rho = 0.05, *p* = 0.5). While not ideal, this observation is not unprecedented [31]. We selected the top 6% of tiles from the first experiment, with similar functional scores, and then re-tested and re-normalized them against the ortholog library with a much greater dynamic range. Both the high reproducibility in our ortholog screen (rho = 0.773–0.959) and the finding that our data from the ortholog screen are highly structured (i.e. similar orthologs show similar activity) support our confidence that our scores are biologically meaningful.

For our initial analyses, we normalized the enrichment scores for all non-human orthologs to the enrichment score of the human ortholog, given that these tiles were first identified on the basis of the human ortholog exhibiting activity (Additional file 2: Table S5). We identified 220 enhancer tiles for which we successfully assayed activity for the human ortholog and at least 14 other orthologs. For each of these orthologs, we asked how well the experimental measurements correlated with the gkm-SVM predictions from Fig. 2c. Specifically, we asked whether the gkm-SVM model predicted functional differences between closely related orthologs by comparing our scores of model predictions vs. functional data (all scores normalized to the human ortholog). There was no correlation between the predicted vs. experimental normalized scores using the MPRA-trained model (Fig. 3b; Spearman rho = − 0.002, *p*-value = 0.892) (Additional file 2: Table S6) or a model trained on HepG2 DNase + H3K4me1 (Spearman rho = − 0.013, *p*-value = 0.633). Therefore, while the kmer-based model performed well at characterizing relative activities of diverse elements (Fig. 2a), it did not predict the relative activities of closely related sequences as measured here (Fig. 3b).

We next performed hierarchical clustering on the vectors of experimentally measured activity for each tile (i.e. where each vector consists of the set of activities experimentally measured for orthologs and ancestral reconstructions of a human-active tile, normalized against the activity of the human ortholog; Fig. 3c). We identified a group of 78 enhancer tiles with relatively higher activity in either humans or hominoids (78/220 or 35.5%) (Fig. 3c, green group), a group of 35 enhancer tiles with relatively lower activity in the Old World monkey lineage (15.9%) (Fig. 3c, orange group), and a group of 52 enhancer tiles with relatively higher activity in the Old World monkey lineage (23.6%) (Fig. 3c, gray group). As a negative control, when we permuted species’ ids for each tile (i.e. shuffling raw scores represented within each column of Fig. 3c, renormalizing, and performing hierarchical clustering), we no longer observe coherent clustering of activity patterns by clades (Additional file 1: Figure S4).

The first group, i.e. the subset of 78 enhancer tiles (35.5% of 220 tested) with greater activity in humans or hominoids relative to other primates, corresponds to the pattern predicted by the ChIP-seq data, slightly higher than the proportion of the 108 tiles (31% of 348 tested) whose computationally predicted activity was concordant with the pattern predicted by the ChIP-seq data (Fig. 2c). However, only 26 enhancer tiles overlapped between these groups, which is not more than expected by chance (*p* = 0.69, Fisher’s exact test). This was consistent with the lack of correlation between the experimental and predicted relative scores shown in Fig. 3b.

For several reasons, we chose to move forward with the experimentally measured activities of primate enhancer tile orthologs. First, we believe that experimental measurements are preferable to computational predictions when available. A condition for this preference is that the experimental measurements are reproducible, which in this case they are (Additional file 1: Figure S3A-C). Second, the computational model used here is predicting the likelihood of a sequence belonging to an active vs. inactive group, while the experimental data measure the relative activity of each sequence. Although improving computational tools remains a paramount goal, experimental data are presently better for quantifying differences in activity, which is the attribute that we would like to correlate with sequence divergence. Third, the differences in experimentally measured activity between orthologs relative to human were generally much greater in magnitude than the computational predictions (e.g. compare Fig. 2c vs. Fig. 3c, which use the same color scale), and furthermore in patterns that were consistent with the phylogeny relating those sequences to one another (Fig. 3c vs. Additional file 1: Figure S4, which is the same data permuted).

#### Evolutionary-functional trajectories for hundreds of enhancer tiles across the primate phylogeny

We had originally normalized enhancer tile activity to the human ortholog with the assumption that most enhancer tiles would be hominoid-specific based on patterns in H3K27ac ChIP-seq data. While our largest group did agree with the ChIP-seq data, it only represented 36% of the tested tiles. Given that the groups that we did observe were relatively coherent in relation to the lineage tree (Fig. 3c), we turned to asking whether we could quantify the enhancer activity of various orthologs relative to their common ancestor.

**Fig. 4:**
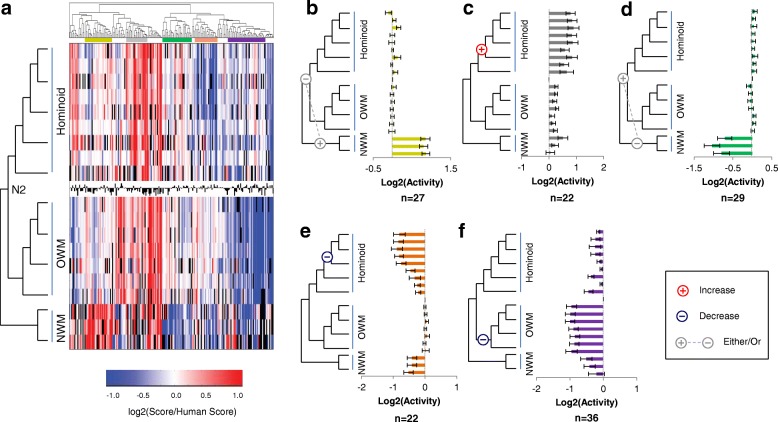
Common Patterns of Enhancer Modulation over the Primate Phylogeny. **a** Functional scores for all enhancer tiles normalized to the MRCA of hominoids and Old World monkeys (N2). Black bar graph in the center contains the N2 score for each tile. Color bars above the heatmap indicate subsets of enhancer tiles exhibiting coherent patterns with respect to gain/loss of activity across the primate phylogeny, including: increased in NWM (yellow), increased in hominoid (gray), decreased in NWM (green), decreased in hominids (orange), and decreased in OWM (purple). **b** The average score normalized to N2 for each species across the group of 27 enhancer tiles with increased activity restricted to the outgroup of New World monkeys. Gray “+” / “-” indicates that there could be either a gain-of-activity event at the “+” or a loss-of-activity event at the “-”. Error bars are one standard error. **c** Same as (**b**) for a group of 22 enhancer tiles with increased activity within the hominoid clade. Red “+” indicates a gain-of-activity event. **d** Same as (**b**) for a group of 29 enhancer tiles with decreased activity restricted to the outgroup of New World monkeys. **e** Same as (**b**) for a group of 22 enhancer tiles with decreased activity in hominids. Blue “-” indicates a loss-of-activity event. **f** Same as (**b**) for a group of 36 enhancer tiles with decreased activity in OWM

For this, we normalized the enhancer tile activity scores for all orthologs to the MRCA of Catarrhines (N2; common ancestor of hominoids and Old World monkeys) (Additional file 2: Table S7). We then performed hierarchical clustering on the 200 enhancer tiles with scores for N2 and at least 14 additional orthologs. The resulting heatmap is shown in Fig. 4a. We observed several subsets of enhancer tiles that exhibited gains or losses in activity as measured by STARR-seq, relative to the experimentally measured activity of the reconstructed sequence of the ancestor to Catarrhines (Additional file 2: Table S8). Many of these subsets were coherent in relation to the lineage tree, meaning that more closely related orthologs exhibited consistent changes in activity in relation to one another.

The first group (yellow; Fig. 4b) contains enhancer tiles with increased activity restricted to the outgroup of New World monkeys. This group contains 27 enhancer tiles (13.5%), and is consistent with either a single loss-of-activity event occurring between the MRCA to Simiformes (hominoids, Old World monkeys, and New World monkeys) and N2, or a single gain-of-activity event on the branch leading to the New World monkeys. As New World monkeys served as our outgroup, we cannot distinguish between these possibilities.

A second group (gray, Fig. 4c) contains 22 enhancer tiles (11%) with increased activity within the hominoid clade. These enhancer tiles can be explained by single gain-of-activity event along the branch from N2 to the MRCA to hominoids. This group of tiles is particularly interesting as a subset of recently evolving primate enhancers, with increased activity unique to hominoids.

A third group (green; Fig. 4d) contains enhancer tiles with decreased activity restricted to the outgroup of New World monkeys. This group contains 29 enhancer tiles (14.5%), and is consistent with either a single gain-of-activity event occurring between the MRCA to Simiformes (hominoids, Old World monkeys, and New World monkeys) and N2, or a single loss-of-activity event on the branch leading to the New World monkeys.

A fourth group (orange; Fig. 4e) contains 22 enhancer tiles (11%) with decreased activity in hominids (great apes and humans) relative to N2. The most parsimonious explanation is a single loss-of-activity event along the branch from the MRCA of hominoids to the MRCA of hominids (Fig. 4e). We do note some decreased activity in NWM, gibbon, N3, and orangutan, but the largest change is localized to hominids. Therefore, while we are highlighting the event on the branch leading to hominids, there were likely additional functional events throughout the tree. This group is particularly interesting in that the human enhancer tiles, which are active based on ChIP-seq and our initial tiling experiment, have lower activity than some ancestral sequences as well as Old and New World monkeys. Looking more broadly, there are 35 enhancer tiles (17%) for which the human sequence exhibits significantly lower activity than the reconstructed N2 ortholog (*p* < 0.05, t-test). This bias towards reductions in activity relative to the ancestral N2 ortholog is not unique to human orthologs. Across the full dataset, 626 orthologs (excluding New World monkeys) showed a significant reduction in activity compared to the N2 ortholog while only 543 showed a significant increase (*p* = 0.0085, two-proportion z-test). This result suggests that the ancestral forms of regulatory sequences queried here tended to have greater activity than descendant sequences.

A fifth group (Fig. 4f) contains enhancer tiles that maintain activity in all orthologs except for Old World monkeys, which have consistently decreased activity relative to N2. It also contains some enhancers with decreased activity in NWM, which may be due to an additional loss-of-activity event. This group contains 36 enhancer tiles (18%). A parsimonious explanation is that this group comprises tiles in which loss-of-activity events occurred on the branch between N2 and the MRCA to Old World monkeys. We also note some decreased activity throughout the tree for this group, but the difference is most pronounced for Old World monkeys.

We examined whether tiles derived from the same enhancer peaks tend to fall within the same groups defined above. The 348 human-active enhancer tiles for which we tested additional orthologs derived from 233 candidate enhancers. Of these 233, 75 contained multiple tiles in our set, 9 of which had pairs of tiles that both fell within one of the five groups, which is significantly greater than expected by chance (*p* < 1e-5, permutation test). Three of these nine pairs of enhancers were overlapping tiles, which can potentially narrow down the location of causal mutation(s).

#### Characterizing molecular mechanisms for enhancer modulation

**Fig. 5:**
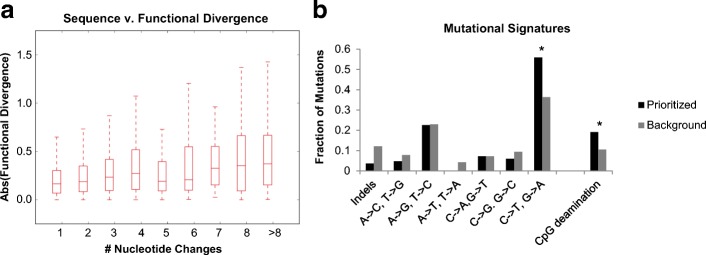
Molecular Characterization of Enhancer Modulation. **a** For every branch along the tree, we calculated the nucleotide and functional divergence. The number of nucleotide changes is on the x-axis and the absolute value of the difference in the logged functional activity between the daughter and ancestral node is on the y-axis. **b** The fraction of indels, A → C and T → G mutations, A → G and T → C mutations, A → T and T → A mutations, C → A and G → T mutations, C → G and G → C mutations, and C → T and G → A mutations in our set of 84 prioritized mutations (those associated with a significant functional difference) in black and 2537 background mutations (those associated with a non-significant functional difference) in gray. Asterisk represents a *p*-value < 0.05 (Fisher’s exact test). First seven tests use a Bonferroni correction. CpG deamination was calculated separately from the mutational spectra, and therefore not corrected for multiple testing

We next explored the relationship between the sequence vs. functional evolution in enhancer activity across the primate phylogeny. As a starting point, we asked whether there was a correlation between the accumulation of sequence variation and the magnitude of change in functional activity for enhancer tiles. For every branch along the tree, we calculated the number of mutations between the mother and daughter nodes and the change in activity between the nodes. There was a significant, albeit modest, correlation between the number of mutations accumulated along a branch and the absolute change in functional activity (Spearman rho = 0.215, *p* = 1.3e-27) (Fig. 5a, Additional file 1: Figure S6). On average, each nucleotide substitution was associated with a 5.4% change in functional activity (slope of best fit line in Additional file 1: Figure S6 normalized to average tile length).

We initially asked whether disruption of certain transcription factor (TF) motifs was associated with changes in functional activity. However, our study was underpowered for this analysis, so we ultimately decided to instead prioritize specific mutations based solely on sequence vs. functional differences across the phylogeny. For each position along a given enhancer tile with a variant in at least one ortholog, we characterized each allele as ancestral (matching the MRCA of human and squirrel monkey, N2) or derived. We then performed Mann-Whitney U tests at each position to test for association between allele status and functional scores, while applying a Bonferroni correction to account for the number of variants tested for each tile. If there were multiple mutations per tile, we only selected the mutation or mutations (in case of ties) with the most significant *p*-values. Through this analysis, we identified a total of 84 mutations that correlated with the functional scores, which we will refer to as “prioritized variants” (Additional file 2: Table S9). Due to the phylogenetic relationship between mutations, some tiles contained multiple prioritized variants, whereas in other cases, we are not calling any significant mutations on a given tile (Additional file 1: Figure S7). We also generated a set of “background variants,” which did not correlate with functional scores (*p* > 0.05, Mann-Whitney U test). Within the 84 prioritized variants, there was a significant overabundance of C → T and G → A mutations over background (*p* = 0.0021, Fisher’s exact test, Bonferroni corrected) (Fig. 5b). In order to test whether this effect is due in part to methylation, we looked at the subset of these C → T and G → A mutations, which disrupted a CpG. Cytosine deamination within a CpG accounted for 19% of our prioritized variants, compared to only 10.5% of background variants (*p* = 0.015, Fisher’s exact test).

## Discussion

While genome-wide studies demonstrate large-scale turnover of enhancers, the general molecular mechanisms underlying this turnover remain largely unexplored. In this study, we characterized modulation in the activity of hundreds of enhancer tiles throughout primate evolution, with nucleotide-level resolution. We first tried to characterize functional changes using computational tools, and although our tools were able to differentiate enhancers with low nucleotide identity (Fig. 2a), they did not correlate well with our ChIP-seq-based predictions (Fig. 2c) and performed poorly at predicting functional changes between evolutionarily similar sequences (Fig. 3b). There are several reasons why computational models might have performed poorly on our data, particularly for predicting *changes* in expression. First, although progress has been made, accurately predicting the effect of nucleotide mutations on gene expression remains a very difficult challenge [36, 37]. Second, since our model was trained on a relatively small sample size (500 positive and 500 negative MPRA sequences), a more limited training set compared to previous attempts at predicting regulatory mutations [36], it will not have seen all combinations of kmers and therefore might miss epistasis between variants and/or TF binding sites. We therefore decided to test all sequences using STARR-seq, a reporter assay that experimentally measures regulatory activity for a library of sequences.

By testing all elements in the same *trans* environment (a single cell type), our experimental approach provided quantitative and directly comparable measurements, allowing us to measure functional differences between closely related sequences. However, this experimental approach assumes conserved trans-environments throughout the primate lineage. Previous studies have indeed noted that both the specificity of transcription factors for DNA and coactivators has remained highly conserved over much longer evolutionary time scales [38–41].

From both our computational predictions and functional scores, we note a low concordance with ChIP-seq based predictions (30–36%). These numbers are similar to previous attempts to replicate biochemical predictions with high-throughput reporter assays [31, 42, 43], and there are plausible explanations for the difference. The first is the inherent contrast between reporter assays and ChIP-seq. While ChIP-seq measures for the presence of biochemical marks associated with enhancer activity, reporter assays directly test sequences of interest for functional activity. However, while ChIP-seq screens these regions in their chromatinized and extended sequence context, traditional reporter assays, as well as the one used here, screen them as short sequences on a plasmid. Chromatin state differences between episomes and native chromosomes may contribute to the differences.

The second explanation relates to the cell and tissue types used. The ChIP-seq predictions were based on experimental data from primary liver samples from three individuals per species. This may contribute to differences for multiple reasons. First, while most non-coding mutations are not expression-modulating [23, 24], we cannot rule out within-species sequence variation between the tissues tested and reference genomes contributing to functional differences. Second, although most of the liver is composed of a single cell type, hepatocytes, there is still more diversity in such primary tissue than in the cell culture system we used for STARR-seq. Moreover, while we maintain a single *trans* environment in testing all orthologs, HepG2 cells are derived from a hepatocellular carcinoma, and likely have acquired changes during cancer development and immortalization, relative to primary liver. However, the fact that our enhancer tiles are both active in HepG2 cells (ChromHMM and STARR-seq) and in primary liver from humans (H3K27ac ChIP-seq) adds to our confidence that we are characterizing bona-fide enhancers.

Through hierarchical clustering of enhancer tiles normalized to human, we identified several functional groups. The largest group matched our ChIP-seq based predictions, with increased activity in humans and/or hominoids compared to other primate orthologs. We also identified a large group with decreased activity in Old World monkeys (concordant with three of the four ChIP-seq based predictions) and a third group with increased activity in Old World monkeys, or decreased activity in humans. The third group is the opposite of what we expected based on our ChIP-seq predictions, and can potentially be accounted for by the explanations above.

We next characterized evolutionary-functional trajectories for 200 of the enhancer tiles by normalizing all orthologs to the MRCA between hominoids and Old World monkeys. We grouped these trajectories using hierarchical clustering, and identified several common patterns of modulation throughout the primate phylogeny. The most common patterns were tiles with a single gain-of-activity in NWM (or loss-of-activity on the branch leading to Catarrhines) (*n* = 27, Fig. 4b), a single gain-of-activity in Hominoids (*n* = 22, Fig. 4c), a single loss-of-activity in NWM (or gain-of-activity on the branch leading to Catarrhines) (*n* = 29, Fig. 4d), a single loss-of-activity in Hominids (*n* = 22, Fig. 4e), and a single loss-of-activity in OWM (*n* = 36, Fig. 4f).

The group of enhancer tiles with decreased activity in hominids may indicate sub-optimization or fine-tuning of enhancers [27]. In total, 17% of our tiles showed a significant reduction of activity in human compared to N2, suggesting that reductions, without complete loss, of activity may in fact be a common phenomenon in primate enhancers. To determine whether sub-optimization was a general trend across the phylogeny, we calculated the number of enhancer tiles with significant increases or decreases in activity relative to N2. We identified significantly more tiles with decreases relative to N2 than increases. All of these findings are concordant with high ancestral activity of present-day enhancers with subsequent loss to fine-tune activity along the phylogeny, at least for the enhancers that we chose to characterize here, which may be biased by the manner in which they were selected.

Ultimately, we wanted to look for general trends between sequence and functional divergence of enhancers throughout evolution. First, we looked at how the number of mutations accumulated along any branch on the tree correlates with the functional divergence along the branch. We found a modest, but significant correlation between sequence and functional divergence (Spearman rho = 0.215, *p* = 1.26e-27). Previous studies have associated naturally occurring genetic variation to evolutionary changes in expression [26] and population variation in expression [23]. Previous studies have also related synthetic variation to changes in reporter activity [20–22, 27, 44]. However, our focus here is on quantifying the relationship between single nucleotide changes between closely related species occurring during neutral evolution and experimentally-measured functional differences.

To further characterize mechanisms of mutations important in enhancer evolution, we utilized the high nucleotide identity between orthologs and reconstructed ancestral sequences to prioritize several variants, whose allele status correlates with functional activity. We relied on prioritizing variants solely based on sequence content and functional scores, resulting in a list of 84 variants which correlated with functional changes. These 84 variants were enriched for cytosine deamination, particularly within CpGs, compared to variants that were not significantly associated with functional scores. Of note, due to the phylogenetic relationship between mutations within each tile, we cannot say with certainty that prioritized mutations are causal. Rather, we are highlighting variants with the most significant *p*-value, analogous to the lead SNP of a GWAS or eQTL study. Additional studies in which each change is evaluated in the context of a common background may be necessary to identify which mutation (or combination of mutations) causally modifies enhancer activity. Especially within closely related species, CpG deamination is a promising source of evolutionary novelty. Since spontaneous deamination of 5-methylcytosine (5mC) yields thymine and G-T mismatch repair is error prone, 5mC has a mutation rate four to fifteen-fold above background [45].

Besides its increased rate of mutation, there are multiple mechanisms by which CpG deamination may play a significant role in enhancer modulation. One mechanism is by introducing novel transcription factor binding sites or disrupting existing binding sites. In fact, Zemojtel et al. suggested that CpG deamination creates TF binding sites more efficiently than other types of mutational events [46]. CpG deamination may also alter enhancer activity by modifying methylation. Enhancer methylation has been correlated with gene expression, most frequently in cancer patients but also in healthy individuals [47]. Notably, enhancer methylation is both correlated with increased and decreased gene expression, possibly explaining why we see an enrichment of CpG deamination in both gain and loss-of-activity events [48].

## Conclusion

In this study, we aimed to characterize general molecular mechanisms that underlie enhancer evolution. In order to do so, we conducted a large-scale screen of enhancer modulation with nucleotide-level resolution by combining genome-wide ChIP-seq with STARR-seq of many orthologs. We characterized evolutionary-functional trajectories for hundreds of enhancer tiles, demonstrating a significant correlation between sequence and functional divergence along the phylogeny. We identify that many present-day enhancers actually have decreased activity relative to their ancestral sequences, supporting the notion of sub-optimization. We prioritized 84 variants, which correlated with functional scores, and found enrichment for cytosine deamination within CpGs among these prioritized events. We propose that CpG deamination may have acted as an important force driving enhancer modulation during primate evolution.

## Methods

### Identification of potential hominoid-specific enhancers

We downloaded processed H3K27ac and H3K4me3 peak calls from Villar et al. [18]. Within each species, we called enhancers as H3K27ac peaks with a mean fold change ≥10 that were not within 1000 bp of an H3K4me3 replicated peak. While the H3K4me3 filter may remove some active enhancers with modest H3K4me3 peaks, it allows us to filter out alternative promoters that may be unannotated in different species. The analysis resulted in 29,139 enhancer calls vs. 29,700 if we include all peaks at least 1 kb away from an annotated TSS (Ensembl v83). We converted all replicated H3K27ac peaks in rhesus, vervet and marmoset to hg19 coordinates using the UCSC liftover tool with a minimum match of 0.5. Villar et al. called the vervet peaks using the rhesus genome as a reference. We identified potential hominoid gain of function enhancers as predicted enhancers that did not have orthologous H3K27ac enrichment within 1 kb from the summit in rhesus, vervet or marmoset. We converted the 10,611 gain of function enhancers back to the marmoset and rhesus genome with a minimum match of 0.9, with 6862 having orthologs in the three genomes. We intersected our 6862 GOF enhancers with ChromHMM strong enhancer calls in HepG2 using bedtools [49], resulting in a final set of 1015 potential hominoid gain of function enhancers predicted to be active in HepG2.

### Design and synthesis of tiles

For each potential hominoid gain of function enhancer, we defined end points by using the intersection of the H3K27ac peak and HepG2 ChromHMM strong enhancer call. For any intersections less than or equal to 200 nt, we designed a 194 bp tile around the center. For intersections with 200 ≤ length ≤ 400, we split the sequence into 3 overlapping fragments. For intersections > 400 nt, we used 100 bp sliding windows. We created negative controls from 800 tiles using uShuffle to create 200 dinucleotide shuffles each [50], and then picked the shuffled sequence with the fewest 7mers present in the original tile. We then synthesized all 10,544 tiles and 800 negative sequences as part of a 244 K 230mer array from Agilent. The library was amplified from the Agilent array using the HSS_cloning-F (5’-TCTAGAGCATGCACCGG-3′) and HSS_cloning-R (5’-CCGGCCGAATTCGTCGA-3′) primers and cloned into the linearized human STARR-seq plasmid using NEBuilder HiFi DNA Assembly Cloning Kit [30]. The library was transformed into NEB C3020 cells and midi-prepped using the ZymoPURE Plasmid Midiprep Kit (Zymo Research).

### Identification of active tiles

We transfected 5μg of our tiling library and 2.5μg of a puromycin expressing plasmid into three 60 mm dishes, each with approximately 1.5 million HepG2 cells using Lipofectamine 3000 (ThermoFisher) according to manufacturer’s instructions. Twenty-four hours post-transfection, we selected cells with 1 ng/mL puromycin for 24 h. Forty-eight hours post transfection, we extracted DNA and RNA from the cells using the Qiagen AllPrep DNA/RNA Mini Kit (Qiagen). We treated RNA with the TURBO DNA-free Kit (ThermoFisher) and performed reverse transcription with SuperScript III Reverse Transcriptase (ThermoFisher). We amplified the cDNA using NEBNext High-Fidelity 2× PCR Master Mix with 5ul of RT reaction with primers HSS-F and HSS-R-pu1 in a 50ul reaction for three cycles with a 65 °C annealing temperature. PCR reactions were cleaned with 1× Agencourt AMPure XP and eluted in 19ul (Beckman Coulter). We then performed a nested PCR using the whole purified cDNA reaction with primers HSS-NFpu1 (5’-CTAAATGGCTGTGAGAGAGCTCAGGGGCCAGCTGTTGGGGTGTCCAC-3′) and pu1R (5’-ACTTTATCAATCTCGCTCCAAACC-3′). DNA was amplified in one reaction using HSS-NFpu1 and HSS-R-pu1 (5’-ACTTTATCAATCTCGCTCCAAACCCTTATCATGTCTGCTCGAAGC-3′) with 1-2μg of DNA in a 50ul reaction and purified with 1.8× AMPure. We added barcodes and Illumina adaptors using Kapa HIFI HotStart Readymix in 50uL reactions with 1ul of previous PCR product with a 65 °C annealing temperature and primers Pu1F-idx (5’-AATGATACGGCGACCACCGAGATCTACACACGTAGGCCTAAATGGCTGTGAGAGAGCTCAG-3′) and Pu1R-idx (5’-CAAGCAGAAGACGGCATACGAGATNNNNNNNNNGACCGTCGGCACTTTATCAATCTCGCTCCAAACC-3′) and sequenced on a 300 cycle NextSeq 500/550 Mid Output v2 kit with PE150bp reads. We aligned sequencing reads to the input library using BWA mem [51]. We then calculated the normalized RNA/DNA ratio (#aligning RNA reads/All RNA reads divided by #aligning DNA reads/All DNA reads) using a hard DNA read cutoff of > 10 and ratio of zero (zero RNA reads) were excluded from analysis. We defined active tiles as ones with a log2 enrichment score > 1.

### Design of orthologs and ancestral sequences

We identified all orthologs using the UCSC liftover tool with a minimum match of 0.9. For each sequence, we determined the longest ortholog, and set it to 194 bp around the center. We then used LiftOver to identify the end points in other species. 348 of the 697 sequences were present through squirrel monkey, and we decided to use squirrel monkey as our outgroup moving forward. For ancestral reconstruction, we trimmed the hg38 phyloP 20way tree to the 11 species of interest and ran the FastML heuristic [35]. We aligned each sequence with ClustalO to obtain a multiple sequence alignment [52], and then ran FastML (v3.1) with default settings on that alignment and the phyloP tree to create ancestral reconstructions.

### Prediction of tiling and evolutionary results

We trained the gksvm-1.2 from Ghandi et al. using an independent dataset (the 500 top- and bottom-scoring lenti-MPRA sequences from Inoue et al. as the positive and negative training sets, respectively), with default settings (Ghandi et al., 2014; Inoue et al., 2016). We used this model to predict scores for all tiles, and calculated the Spearman rho with our functional data. We next predicted scores for our positive human tiles and predicted negative orthologs from rhesus, vervet and marmoset and performed a two-sample t-test for each comparison. We calculated delta gkm-SVM scores by subtracting the predicted score of each ortholog from the predicted score of the human ortholog (in log scale). We then predicted all eleven orthologs and nine ancestral nodes for all 348 enhancers.

### Functional testing of orthologs and ancestral sequences

All orthologs and ancestral sequences were synthesized as part of an Agilent 230mer 244 K array. We appended 5 bp degenerate barcodes to each sequence by amplifying off the array with JK_R48_5N_HSSR (5’-CCGGCCGAATTCGTCGANNNNNCCATTGAGCACGACAGC-3′) and HSS_cloning-F (5’-TCTAGAGCATGCACCGG-3′). We then cloned the library into the STARR-seq vector in NEB C3020 cells, transfected into HepG2 cells, and prepared sequencing libraries as described above. Since some orthologs have very similar sequences, we aligned sequencing reads to our reference and only extracted error-free matches. For each barcode-tile pair, we calculated the #aligning RNA reads/Total RNA reads divided by the #aligning DNA reads/Total DNA reads. We then took the log2 of this ratio for each barcode-tile pair, and averaged all pairs for each tile. We used a hard cutoff of 10 DNA reads for any barcode-tile pair, and ratio of zero (zero RNA reads) were excluded from analysis before log transformation. Hierarchical clustering was performed on the top ten principal components (to handle missing values) with SciPy v0.19.1 with Python v2.7.3 using the distance metric set as cosine (scipy.spatial.distance.pdist) and the linkage method set as average (scipy.cluster.hierarchy.linkage).

### Molecular characterization

We first looked to see whether the turnover of any transcription factor motifs correlated with functional scores. We ran FIMO to identify TF motifs from HOCOMOCO v9 that were lost or gained in at least one ortholog for each enhancer [32, 33]. For one enhancer at a time, we ran a linear regression for the presence or absence of each TF motif against the functional scores of all orthologs tested. For each TF, we then tested whether the mean slope across all enhancers was equal to zero using a two-sample t-test.

We next looked to see whether any sequence mutations in an enhancer correlated with functional scores of the orthologs. For each enhancer, we performed a multiple sequence alignment using ClustalO. For each site along the enhancer (skipping the first to avoid alignment artifacts), we characterized the allele as ancestral or derived. For each site with a singleton derived allele in at least one ortholog, we conducted a Mann-Whitney U test to see whether the allele associated with the functional scores. We then corrected the *p*-values for the number of sites tested along the enhancer using a Bonferroni correction. For enhancer tiles with multiple prioritized mutations, we only included the mutations with the most significant p-value (if there was a tie, we included all with the same p-value). We then characterized the nucleotide change and summed the number of events over all enhancers. We calculated the Fisher’s exact *p*-value for each type of mutational event, using Bonferroni’s correction to adjust for multiple hypothesis testing. We then looked to see what fraction of C → T and G → A mutations disrupted CpGs, and calculated the Fisher’s exact *p*-value.

## Additional files

Additional file 1: **Figure S1.** Tiling Across Large Enhancer Regions. **Figure S2.** Reproducibility of Tiling Scores. **Figure S3.** Reproducibility of Functional Scores for Orthologs. **Figure S4.** Permuted Species’ IDs. **Figure S5.** Confidence of Ancestral Reconstructions. **Figure S6.** Sequence vs. Functional Divergence. **Figure S7.** Number of Prioritized Mutations per Tile. (DOCX 514 kb)

Additional file 2: **Table S1.** Tiling Scores. **Table S2.** TF motif enrichment. **Table S3.** gkm-SVM Prediction of Tiles. **Table S4.** Ortholog Scores. **Table S5.** Orthologs normalized to Human. **Table S6.** gkm-SVM Predictions of Orthologs. **Table S7.** Orthologs normalized to N2. **Table S8.** Enhancer Groups from Fig. 4. **Table S9.** Prioritized Mutations. (XLSX 974 kb)

## References

[1] Britten RJ, Davidson EH (1971). Repetitive and non-repetitive DNA sequences and a speculation on the origins of evolutionary novelty. Q Rev Biol.

[2] King M, Wilson AC (1975). Evolution at two levels in humans and chimpanzees. Science.

[3] Banerji J, Rusconi S, Schaffner W (1981). Expression of a beta-globin gene is enhanced by remote SV40 DNA sequences. Cell.

[4] Moreau P, Hen R, Wasylyk B, Everett R, Gaub MP, Chambon P (1981). The SV40 72 base repair repeat has a striking effect on gene expression both in SV40 and other chimeric recombinants. Nucleic Acids Res.

[5] Wray GA. The evolutionary significance of cis-regulatory mutations. Nat Rev Gen. 2007;8:206–17. 10.1038/nrg2063. 10.1038/nrg2063 17304246

[6] True JR, Carroll SB (2002). Gene co-option in physiological and morphological evolution. Annu Rev Cell Dev Biol.

[7] Frankel N, Davis GK, Vargas D, Wang S, Stern DL (2010). Phenotypic robustness conferred by apparently redundant transcriptional enhancers. Nature.

[8] Levine M (2010). Transcriptional enhancers in animal development and evolution. Curr Biol.

[9] Wittkopp PJ, Kalay G (2011). Cis -regulatory elements : molecular mechanisms and evolutionary processes underlying divergence. Nat Rev Genet.

[10] Wittkopp PJ, True JR, Carroll SB. Reciprocal functions of the Drosophila yellow and ebony proteins in the development and evolution of pigment patterns. Development. 2002;1858:1849–58. 10.1242/dev.129.8.1849 11934851

[11] Gompel N, Prud B, Wittkopp PJ, Kassner VA, Carroll SB (2005). Chance caught on the wing : cis -regulatory evolution and the origin of pigment patterns in Drosophila.

[12] Chan YF, Marks ME, Jones FC, Jr GV, Shapiro MD, Brady SD, Southwick AM, Absher DM, Grimwood J, Schmutz J, Myers RM, Petrov D, Jónsson B, Schluter D, Bell MA, Kingsley DM (2010). Adaptive evolution of pelvic reduction of a Pitx1 enhancer. Science.

[13] Bersaglieri T, Sabeti PC, Patterson N, Vanderploeg T, Schaffner SF, Drake JA, Rhodes M, Reich DE, Hirschhorn JN (2004). Genetic signatures of strong recent positive selection at the lactase gene. Am J Hum Genet.

[14] Tishkoff SA, Reed FA, Ranciaro A, Voight BF, Babbitt CC, Silverman JS, Powell K, Mortensen HM, Hirbo JB, Osman M, Ibrahim M, Omar SA, Lema G, Nyambo TB, Ghori J, Bumpstead S, Pritchard JK, Wray GA, Deloukas P (2007). Convergent adaptation of human lactase persistence in Africa and Europe. Nat Genet.

[15] Kunarso G, Chia N, Jeyakani J, Hwang C, Lu X, Chan Y, Ng H, Bourque G (2010). Transposable elements have rewired the core regulatory network of human embryonic stem cells. Nat Genet.

[16] Mikkelsen TS, Xu Z, Zhang X, Wang L, Gimble JM, Lander ES, Rosen ED (2010). Comparative Epigenomic analysis of murine and human Adipogenesis. Cell.

[17] Cotney J, Leng J, Yin J, Reilly SK, Demare LE, Emera D, Ayoub AE, Rakic P, Noonan JP (2013). The evolution of lineage-specific regulatory activities in the human embryonic limb. Cell.

[18] Villar D, Berthelot C, Flicek P, Odom DT, Villar D, Berthelot C, Aldridge S, Rayner TF, Lukk M, Pignatelli M (2015). Enhancer evolution across 20 mammalian species article enhancer evolution across 20 mammalian species. Cell.

[19] Trizzino M, Park Y, Holsbach-beltrame M, Aracena K. Transposable elements are the primary source of novelty in primate gene regulation. Genome Res. 2017;27(10):1623-33. 10.1101/gr.218149.116 PMC5630026 28855262

[20] Patwardhan RP, Lee C, Litvin O, Young DL, Pe D, Shendure J (2009). High-resolution analysis of DNA regulatory elements by synthetic saturation mutagenesis. Nat Biotechnol.

[21] Patwardhan RP, Hiatt JB, Witten DM, Kim MJ, Smith RP, May D, Lee C, Andrie JM, Lee S-I, Cooper GM, Ahituv N, Pennacchio LA, Shendure J (2012). Massively parallel functional dissection of mammalian enhancers in vivo. Nat Biotechnol.

[22] Melnikov A, Murugan A, Zhang X, Tesileanu T, Wang L, Rogov P, Feizi S, Gnirke A, Callan CG, Kinney JB, Kellis M, Lander ES, Mikkelsen TS (2012). Systematic dissection and optimization of inducible enhancers in human cells using a massively parallel reporter assay. Nat Biotechnol.

[23] Vockley CM, Guo C, Majoros WH, Nodzenski M, Scholtens DM, Hayes MG, Lowe WL, Reddy TE (2015). Massively parallel quantification of the regulatory effects of noncoding genetic variation in a human cohort. Genome Res.

[24] Tewhey R, Kotliar D, Park DS, Liu B, Winnicki S, Steven K (2016). Direct identification of hundreds of expression-modulating variants using a multiplexed reporter assay. Cell.

[25] Ulirsch JC, Nandakumar SK, Wang L, Giani FC, Rogov P, Melnikov A, Mcdonel P, Do R, Tarjei S (2016). Systematic functional dissection of common genetic variation affecting red blood cell traits. Cell.

[26] Arnold CD, Gerlach D, Spies D, Matts JA, Sytnikova YA, Pagani M, Lau NC, Stark A (2014). Quantitative genome-wide enhancer activity maps for five Drosophila species show functional enhancer conservation and turnover during cis-regulatory evolution. Nat Genet.

[27] Farley EK, Olson KM, Zhang W, Brandt AJ, Rokhsar DS, Levine MS (2015). Suboptimization of developmental enhancers. Science.

[28] Villar D, Berthelot C, Aldridge S, Rayner TF, Lukk M, Pignatelli M, Park TJ, Deaville R, Erichsen JT, Jasinska AJ, Turner JMA, Bertelsen MF, Murchison EP, Flicek P, Odom DT. Enhancer evolution across 20 mammalian species. ArrayExpress. 2015; https://www.ebi.ac.uk/arrayexpress/experiments/E-MTAB-2633/. 10.1016/j.cell.2015.01.006 PMC4313353 25635462

[29] Ernst J, Kheradpour P, Mikkelsen TS, Shoresh N, Ward LD, Epstein CB, Zhang X, Wang L, Issner R, Coyne M, Ku M, Durham T, Kellis M, Bernstein BE (2011). Mapping and analysis of chromatin state dynamics in nine human cell types. Nature.

[30] Arnold CD, Gerlach D, Stelzer C, Boryn LM, Rath M, Stark A, Boryń ŁM, Rath M, Stark A (2013). Genome-wide quantitative enhancer activity maps identified by STARR-seq. Science.

[31] Inoue F, Kircher M, Martin B, Cooper GM, Witten DM, Mcmanus MT, Ahituv N, Shendure J (2016). A systematic comparison reveals substantial differences in chromosomal versus episomal encoding of enhancer activity. Genome Res.

[32] Kulakovskiy IV, Medvedeva YA, Schaefer U, Kasianov AS, Vorontsov IE, Bajic VB, Makeev VJ (2012). HOCOMOCO : a comprehensive collection of human transcription factor binding sites models. Nucleic Acids Res.

[33] Grant CE, Bailey TL, Noble WS (2011). FIMO : scanning for occurrences of a given motif. Bioinformatics.

[34] Ghandi M, Lee D, Mohammad-noori M, Beer MA. Enhanced regulatory sequence prediction using gapped k-mer features. PLoS Comput Biol. 2014;10 10.1371/journal.pcbi.1003711. 10.1371/journal.pcbi.1003711 PMC4102394 25033408

[35] Ashkenazy H, Penn O, Doron-Faigenboim A, Cohen O, Cannarozzi G, Zomer O, Pupko T (2012). FastML: a web server for probabilistic reconstruction of ancestral sequences. Nucleic Acids Res.

[36] Lee D, Gorkin DU, Baker M, Strober BJ, Asoni AL, Mccallion AS, Beer MA (2015). A method to predict the impact of regulatory variants from DNA sequence. Nat Genet.

[37] Beer MA (2017). Predicting enhancer activity and variant impact using. Hum Mutat.

[38] Dowell RD (2010). Transcription factor binding variation in the evolution of gene regulation. Trends Genet.

[39] Zheng W, Gianoulis TA, Karczewski KJ, Zhao H, Snyder M (2011). Regulatory variation within and between species. Annu Rev Genomics Hum Genet.

[40] Nitta KR, Jolma A, Yin Y, Morgunova E, Kivioja T, Akhtar J, Hens K, Toivonen J, Polytechnique F. Conservation of transcription factor binding specificities across 600 million years of bilateria evolution. Elife. 2015:1–20. 10.7554/eLife.04837. 10.7554/eLife.04837 PMC4362205 25779349

[41] Long HK, Prescott SL, Wysocka J (2016). Review ever-changing Landscapes : transcriptional enhancers in development and evolution. Cell.

[42] Kheradpour P, Ernst J, Melnikov A, Rogov P, Wang L, Alston J, Mikkelsen TS, Kellis M (2013). Systematic dissection of regulatory motifs in 2000 predicted human enhancers using a massively parallel reporter assay.

[43] Kwasnieski JC, Fiore C, Chaudhari HG, Cohen BA (2014). High-throughput functional testing of ENCODE segmentation predictions. Genome Res.

[44] Smith RP, Taher L, Patwardhan RP, Kim MJ, Inoue F, Shendure J, Ovcharenko I, Ahituv N (2013). Massively parallel decoding of mammalian regulatory sequences supports a flexible organizational model. Nat Genet.

[45] Cooper DN, Mort M, Stenson PD, Ball EV, Chuzhanova NA (2010). Methylation-mediated deamination of 5-methylcytosine appears to give rise to mutations causing human inherited disease in CpNpG trinucleotides , as well as in CpG dinucleotides. Hum Genomics.

[46] Zemojtel T, Kielbasa M, Szymin PF, Arndt S, Behrens G, Bourque MV (2011). CpG deamination creates transcription factor – binding. Genome Biol Evol.

[47] Aran D, Hellman A (2013). DNA methylation of transcriptional enhancers and Cancer predisposition. Cell.

[48] Long MD, Smiraglia DJ, Campbell MJ. The genomic impact of DNA CpG methylation on gene Expression ; relationships in prostate Cancer. Biomol Ther. 2017;7 10.3390/biom7010015. 10.3390/biom7010015 PMC5372727 28216563

[49] Quinlan AR, Hall IM (2010). BEDTools : a flexible suite of utilities for comparing genomic features. Bioinformatics.

[50] Jiang M, Anderson J, Gillespie J, Mayne M. uShuffle : a useful tool for shuffling biological sequences while preserving the k-let counts. BMC Bioinformatics. 2008;9 10.1186/1471-2105-9-192. 10.1186/1471-2105-9-192 PMC2375906 18405375

[51] Li H. Aligning sequence reads , clone sequences and assembly contigs with BWA-MEM. arXiv. 2013;27:1623-33.

[52] Sievers F, Wilm A, Dineen D, Gibson TJ, Karplus K, Li W, Lopez R, Thompson JD, Higgins DG, Mcwilliam H, Remmert M, Söding J. Fast , scalable generation of high-quality protein multiple sequence alignments using Clustal omega. Mol Syst Biol. 2011;7 10.1038/msb.2011.75. 10.1038/msb.2011.75 PMC3261699 21988835

[53] Klein JC, Keith A, Agarwal V, Durham TJ, Shendure J. Functional characterization of enhancer evolution in the primate lineage. GEO. 2018; https://www.ncbi.nlm.nih.gov/geo/query/acc.cgi?acc=GSE113978. 10.1186/s13059-018-1473-6 PMC6060477 30045748

[54] Kent WJ, Sugnet CW, Furey TS, Roskin KM, Pringle TH, Zahler AM, Haussler D (2002). The human genome browser at UCSC. Genome Res.

