# Supplementary material for: Functional characterization of enhancer evolution in the primate lineage
Source: Genome Biol. 2018 Jul 25;19:99. doi: 10.1186/s13059-018-1473-6 (PMC6060477; doi:10.1186/s13059-018-1473-6)
Supplement: Supplementary file 1 — Figure S1. Tiling Across Large Enhancer Regions. Figure S2. Reproducibility of Tiling Scores. Figure S3. Reproducibility of Functional Scores for Orthologs. Figure S4. Permuted Species’ IDs. Figure S5. Confidence of Ancestral Reconstructions. Figure S6. Sequence vs. Functional Divergence. Figure S7. Number of Prioritized Mutations per Tile. (DOCX 514 kb) [file 13059_2018_1473_MOESM1_ESM.docx]

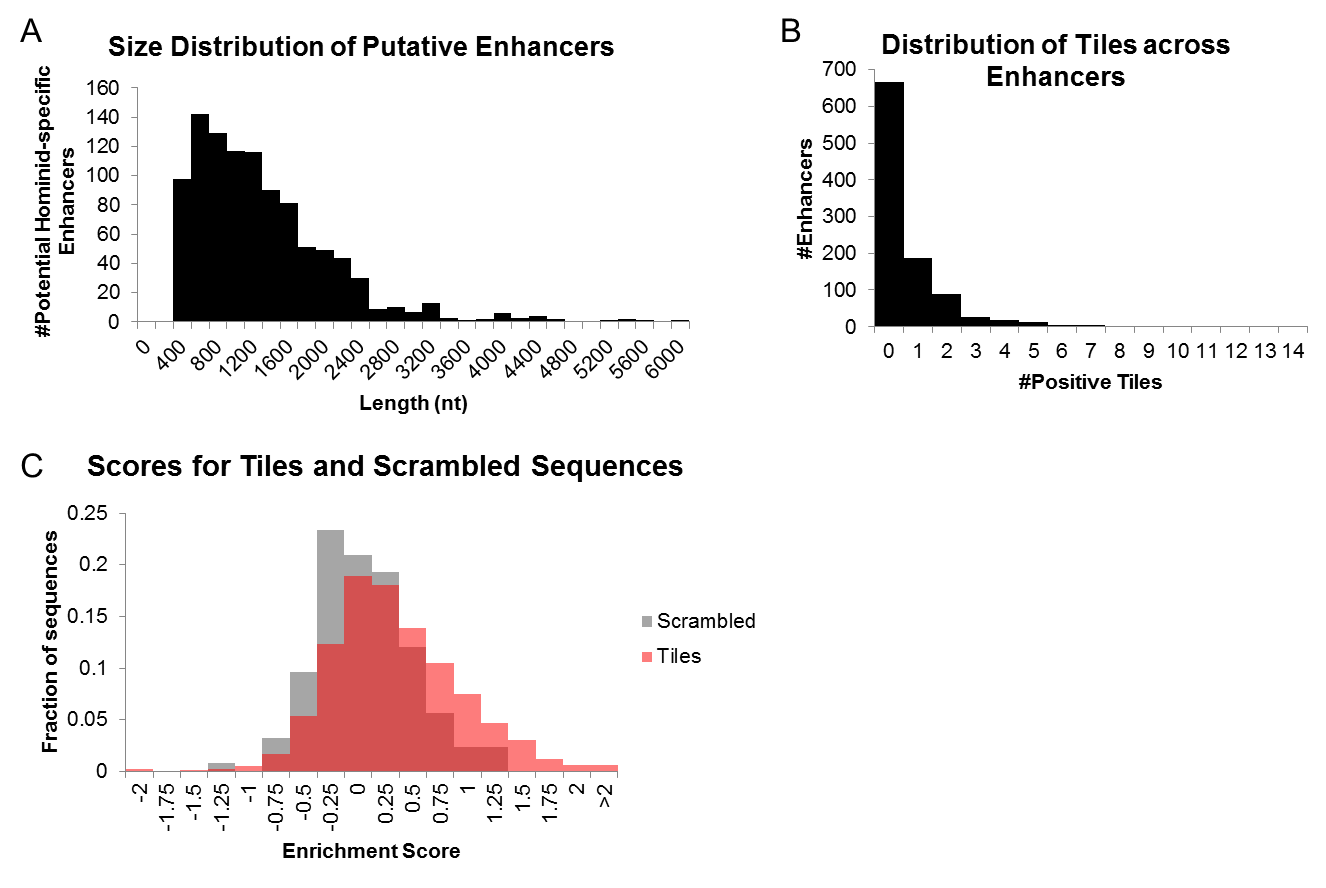


Fig. S1. **Tiling Across Large Enhancer Regions.** A) Histogram of the size for each putative hominoid-specific gain-of-function enhancer defined by the intersection of H3K27ac ChIP-seq from primary tissue and HepG2 ChromHMM strong-enhancer calls. B) Histogram of the number of positive tiles (defined as an enrichment score greater than 2) per putative enhancer. C) Histogram of Z-scores for 6,724 tiles and 124 scrambled sequences.


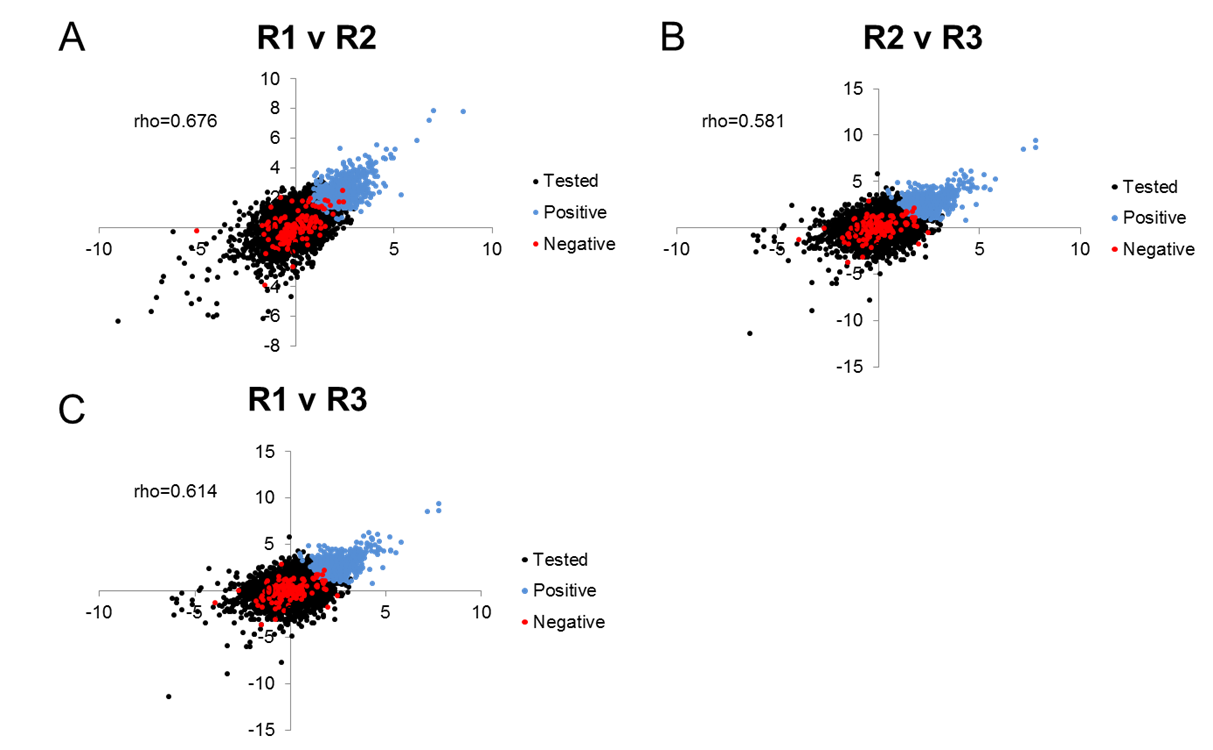


Fig. S2. **Reproducibility of Tiling Scores.** A) Correlation between replicates 1 and 2. Blue points are over 2 standard deviations higher than the average scrambled sequence. Red points are the scrambled sequences (negative controls). Black points are all other tested sequences. B) Correlation between replicates 2 and 3 C) Correlation between replicates 1 and 3.


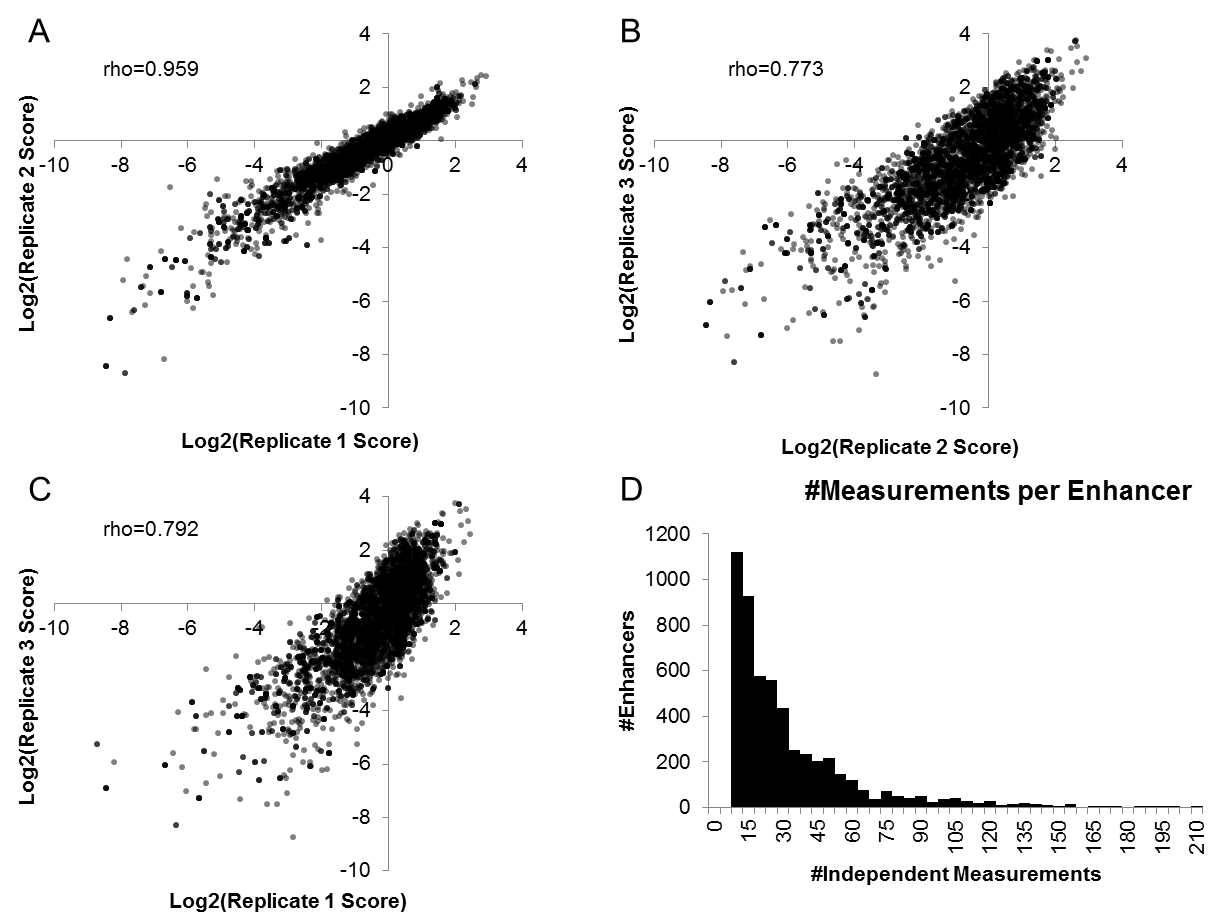


Fig. S3. **Reproducibility of Functional Scores for Orthologs.** A) Spearman correlation between log_2_(normalized RNA/DNA) for biological replicates 1 and 2. B) Spearman correlation between replicates 2 and 3. C) Spearman correlation between replicates 1 and 3. D) Histogram of the number of independent measurements (barcodes) for each enhancer summed across all three replicates.


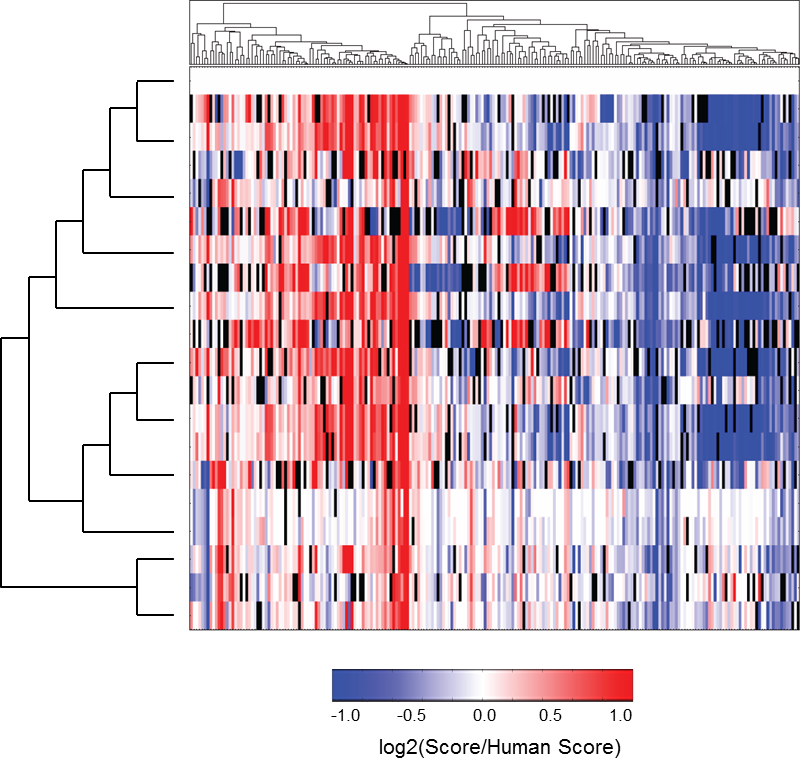


Fig. S4. **Permuted Species’ IDs.** Species’ IDs were randomly permuted (seed=40) before normalization. Orthologs were hierarchically clustered and the same heatmap from Figures 2C and 3C was created.


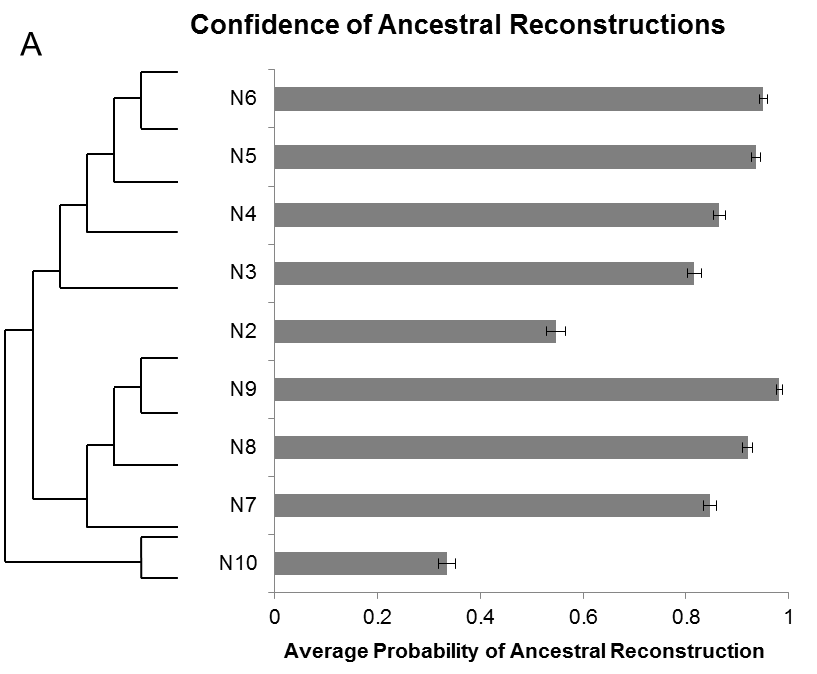


Fig. S5. **Confidence of Ancestral Reconstructions.** The average marginal probability for each ancestral node across all 348 enhancers. Error bars indicate one standard error around the average probability for each node.


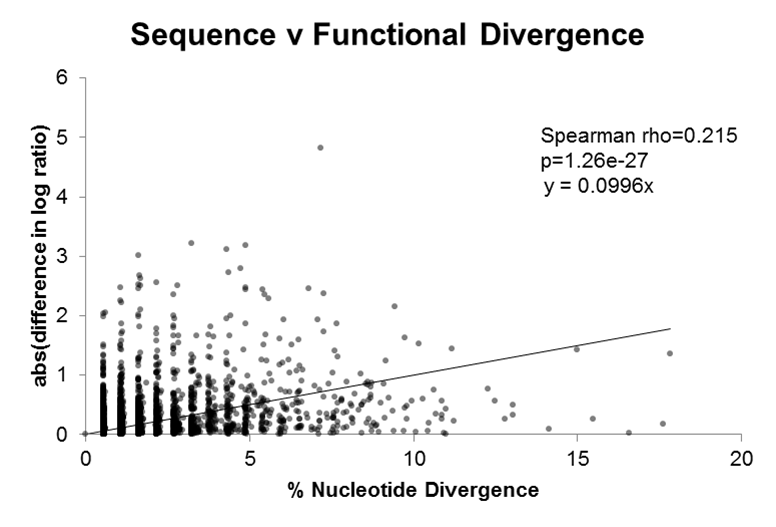


Fig. S6. **Sequence vs. Functional Divergence.** The absolute value of the difference in functional score along a branch v the percent nucleotide divergence between the mother and daughter. This is the same data plotted in Figure 5A. The best fit line is shown, with the intercept set to 0. Average functional change per nucleotide was calculated by taking the slope of the best fit line normalized to the average length of an enhancer tile (185 bp).


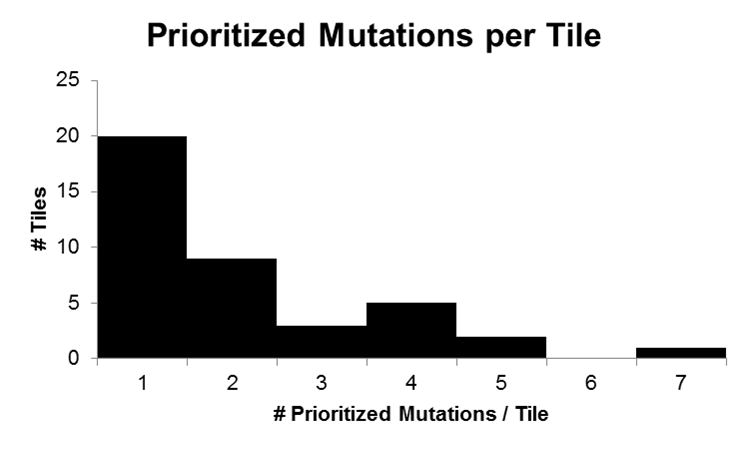


Fig. S7. **Number of Prioritized Mutations per Tile.** The number of prioritized mutations per tile. For cases with more than one, all mutations have the same p-value. 40 of the tiles had at least one prioritized mutation (shown in plot), while 308 tiles had no prioritized mutations. Many of the 308 had missing orthologs and therefore decreased power to detect correlations.
